# Monitoring carotid arterial stiffness using non-contrast-enhanced 4D MR angiography

**DOI:** 10.1186/s41747-026-00716-z

**Published:** 2026-05-06

**Authors:** Isabel Montón Quesada, Thomas Baumgartner, Augustin C. Ogier, Jean-Baptiste Ledoux, Robin Ferincz, Jérôme Yerly, Matthias Stuber, Christopher W. Roy, Lorenz Hirt, Ruud B. van Heeswijk

**Affiliations:** 1 Department of Radiology, Lausanne University Hospital (CHUV) and University of Lausanne (UNIL), Lausanne, Switzerland; 2 Neurology Service, Cerebrovascular Center, Lausanne University Hospital (CHUV), Lausanne, Switzerland; 3 CIBM Center for Biomedical Imaging, Lausanne, Switzerland

**Keywords:** Carotid arteries, Elastic modulus, Magnetic resonance angiography, Ultrasound, Vascular stiffness

## Abstract

**Objective:**

The anatomy of carotid arteries is evaluated with magnetic resonance angiography (MRA), while its stiffness-related parameters are evaluated using ultrasound. An isotropic three-dimensional (3D) MRA technique enabling the measurement of diameter changes could provide a dynamic assessment of the entire carotid tree, extending the existing clinical carotid MR toolset. The goal of this feasibility study was to comprehensively characterize stiffness-related parameters with dynamic 3D MRA and evaluate its repeatability compared to ultrasound.

**Materials and methods:**

A non-contrast-enhanced free-running 3D radial MRA sequence was used in 18 healthy volunteers (9 males, 9 females, aged 49 ± 20 years, mean ± standard deviation), scanned twice with both MRA and ultrasound. Diameter changes throughout the cardiac cycle were measured in MRA and ultrasound images to compute stiffness-related parameters: systolic-diastolic relative diameter change (RDC), stiffness index, arterial compliance (AC), local pulse wave velocity and pressure–strain Young’s elastic modulus (E), computed for the left (LC) and right (RC) common carotid arteries. Repeatability was evaluated for systolic and diastolic diameter measurements using the intraclass correlation coefficient (ICC). Sub-analyses were performed on a junior and senior subgroup of the volunteers.

**Results:**

When evaluating MRA *versus* ultrasound differences, a relative MRA overestimation of the RDC in the senior cohort (*p* < 0.001) led to higher AC (*p* = 0.010) and E (*p* = 0.042). All stiffness-related parameters were significantly different between the age cohorts. MRA showed high repeatability for the LC (ICC = 0.95) and RC (ICC = 0.93) compared to ultrasound (ICC = 0.78, ICC = 0.51, respectively).

**Conclusion:**

Carotid stiffness can be repeatably quantified with non-contrast-enhanced 4D MRA, providing information traditionally obtained with ultrasound.

**Relevance statement:**

Dynamic free-running non-contrast-enhanced MRA allows the noninvasive quantification of carotid dynamic properties and anatomy, demonstrating its potential for the assessment of 3D vascular age, showing superior repeatability compared to the clinical standard ultrasound.

**Key Points:**

- Carotid stiffness is assessed with ultrasound and anatomy with MR imaging; combining both assessments in an examination would be valuable.
- Carotid stiffness can be repeatably quantified with free-running non-contrast-enhanced MRA and yields metrics comparable to ultrasound in healthy volunteers.
- Dynamic 3D MRA may enable the measurement of stiffness parameters throughout the carotid tree.

**Graphical Abstract:**

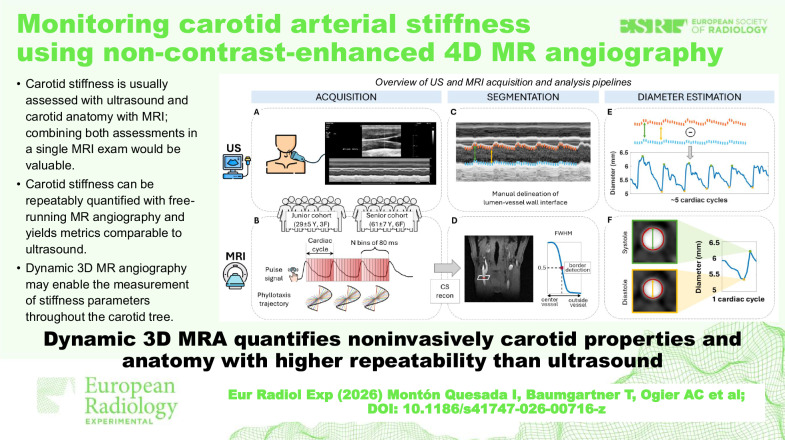

## Background

Stroke is one of the leading causes of disability worldwide and is often related to atherosclerotic plaque rupture in the carotid arteries in the neck [1]. Besides clinical symptoms, current carotid plaque treatment decisions are mostly based on the degree of stenosis and whether the stenosis is symptomatic or not [2]. However, changes in dynamic properties such as vessel wall stiffness are also prevalent in patients with plaque rupture risk [3]. Furthermore, processes such as atherosclerosis, increased arterial stiffness and vessel wall thickening can lead to carotid diameter enlargement, which has been linked to stroke and the degree of atherosclerotic plaque [4]. As arterial stiffness progresses with age, the precise relationship between stroke and carotid stiffness remains a topic of high research interest [5]. The stiffness index of the carotid artery is highly correlated with the local atherosclerotic burden and may help inform clinical decision-making in asymptomatic subjects [6]. An increased arterial stiffness correlates with a higher likelihood of carotid intraplaque hemorrhage, which is an established marker of vulnerable plaque and increased risk of adverse cardiovascular events [7]. Therefore, arterial stiffness is a highly relevant clinical biomarker for the assessment of the severity of carotid artery atherosclerosis, likelihood of intraplaque hemorrhage, and associated complications.

To quantify the degree of atheromatous stenosis in the carotid artery (a static measurement), magnetic resonance angiography (MRA) is commonly used [8]. However, ultrasound is routinely employed to determine the degree of carotid stenosis based on multiple parameters, including blood flow velocity measurements at the site of stenosis and the presence or absence of remote hemodynamic changes [9]. Ultrasound can also be used to dynamically track vessel diameter changes between the systolic and diastolic cardiac phases (*i.e*., the systolic-diastolic relative diameter change, RDC). Typically, quantitative parameters such as the stiffness index β, which reflects the arterial capacity to expand and contract with variations in blood pressure, and the elastic modulus, which quantifies the arterial resistance to deformation, are employed to assess arterial stiffness [3, 10–13]. The main drawback of the ultrasound imaging techniques used to measure these variables, such as M-mode and echo-tracking, is that they are usually two-dimensional (2D), as they only provide a one-dimensional (1D) view of the structures along one ultrasound line through time. Nevertheless, such dynamic biomarkers would be a useful complement to the static three-dimensional (3D) MRA toolset [11].

In magnetic resonance imaging (MRI) at 3 T, gradient-recalled echo sequences are commonly used instead of high-contrast balanced steady-state free precession bSSFP imaging due to their low susceptibility to magnetic field inhomogeneities [14]. However, standard gradient-recalled echo images lack contrast between blood and its surrounding structures. To compensate for this, gadolinium-based contrast agents and time-of-flight imaging [8] are frequently used. Although gadolinium-based contrast agents are only an issue in limited patient populations (*e.g*., patients with advanced kidney disease) [15], contrast-agent-free imaging is also of interest because it can help shorten and simplify clinical workflows, thereby reducing examination costs as well as water contamination [16].

Among the various sequences available for dynamic MRI, free-running (FR) acquisitions, originally developed for cardiac imaging [17, 18], are particularly interesting for the carotid arteries. These continuous, ungated acquisitions allow for retrospective motion-resolved reconstruction of 3D, four-dimensional (4D) imaging (3D + cardiac phases), or even five-dimensional (5D) imaging (4D + respiratory phases), which may also be of use to capture the dynamic vascular behavior in the carotid arteries.

We therefore implemented a FR gradient-recalled echo technique with slab-selective water-excitation radiofrequency (RF) pulses to generate inflow contrast while simultaneously suppressing the fat signal. Here, blood flowing in from outside the excited slab generates a higher signal intensity as it has not been previously saturated like stationary tissue.

The objective of this study was to assess the feasibility and performance of 4D FR dynamic MRA for the quantification of arterial stiffness and carotid diameter changes throughout the cardiac cycle without the need for contrast agents and compare it to the clinical standard ultrasound technique.

## Methods

### Study cohort

This study included 18 healthy volunteers (9 females), aged 49 ± 20 years (mean ± standard deviation), ranging 23–78 years, weight 70 ± 11 kg, body mass index 23 ± 2, and without known cardiovascular disease. All experiments were approved by the local ethics committees (Ethics Committee of the Canton of Vaud under number 2021-00697), and written and informed consent was given by all participants. These participants were also divided into two subgroups for secondary analyses. The first subgroup, referred to as the ‘junior’ cohort, included 9 volunteers (3 females; age 29 ± 5 years; range 23–42 years, weight 73 ± 12 kg, body mass index 24 ± 2). The second subgroup, referred to as the ‘senior’ cohort, comprised 9 volunteers (6 females; age 61 ± 7 years; range 58–78 years, weight 66 ± 8 kg, body mass index 23 ± 3).

### Ultrasound protocol

**Fig. 1:**
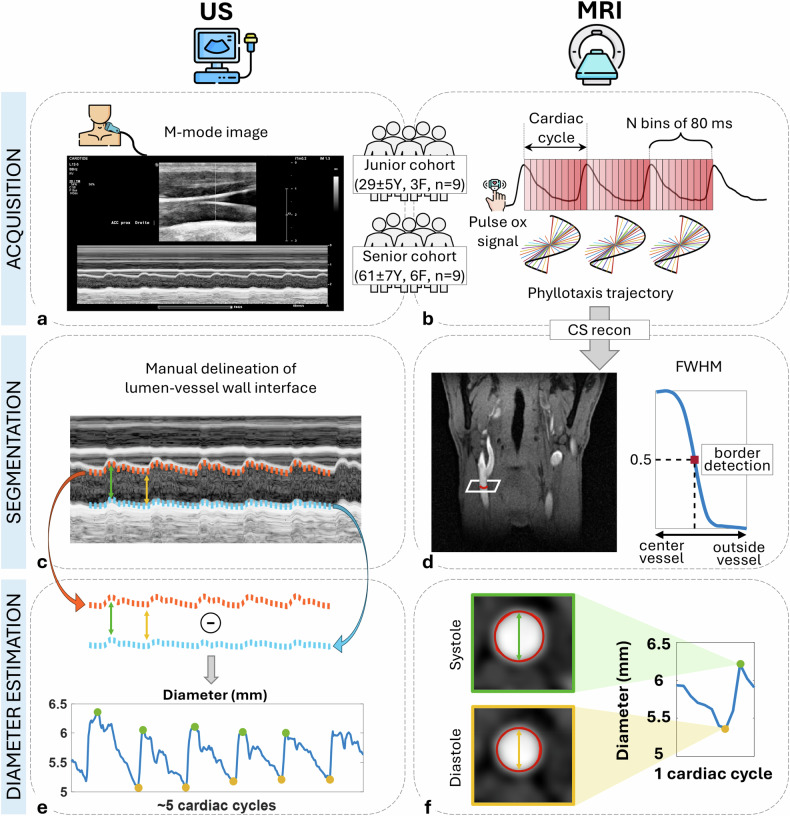
Overview of ultrasound and magnetic resonance angiography (MRA) acquisition and analysis pipelines. **a** Acquisition of M-mode ultrasound images and (**b**) continuous acquisition of MRA data using a segmented three-dimensional golden-angle top-to-bottom phyllotaxis trajectory and k-space cardiac binning into *N* bins of 80 ms using the pulse oximeter signal for two cohorts (junior and senior). **c** Manual delineation of the interface between the vessel wall and the blood pool in ultrasound. **d** Semi-automatic detection of the vessel wall based on the full-width at half-maximum (FWHM), defined as the width between two points at half the signal range. **e** For ultrasound, computation of the systolic (yellow dots) and diastolic (green dots) diameters from the previously delineated boundaries. **f** Detection of the systolic and diastolic diameters in MRA

Participants were scanned with an ultrasound system (EPIQ Elite, Philips) with a 12 MHz L12-3 linear array probe, after 10 min of supine rest with their head and torso slightly tilted up. M-mode images (Fig. 1a) were acquired at 250 frames per second with a 0.1 mm spatial resolution, perpendicular to the carotid artery walls, 1–2 cm below the carotid bifurcation and during 5 consecutive heartbeats. Both the left (LC) and right (RC) common carotid arteries were scanned twice for repeatability assessments in a subset of 14 participants (7 females; age 55 ± 19 years; range 23–78 years, weight 68 ± 9 kg) due to scan time constraints. Brachial pressures were also measured before and after the ultrasound scans in a supine position with an oscillometric semi-automatic sphygmomanometer (VS-900, Mindray Medical International Limited) and averaged afterwards.

### MR protocol

All data were acquired on a 3-T clinical scanner (MAGNETOM PrismaFit, Siemens Healthineers) with a FR gradient-recalled echo sequence that continuously sampled k-space using a 3D golden-angle top-to-bottom radial phyllotaxis trajectory [19, 20] (Fig. 1b). Acquisition parameters were chosen as a compromise between signal-to-noise ratio and acquisition time and included: acquired and reconstructed isotropic voxel size (0.6 mm)^3^, field of view 160 mm in all directions, RF excitation angle α = 8°, repetition time/echo time 5.97 ms/2.72 ms, pixel bandwidth 543 Hz/pixel. Acquisition time was a fixed 10:03 min:s. For inflow contrast generation, slab-selective binomial water-excitation sinc-shaped RF pulses [21] were used in the axial orientation to excite half of the cubic field of view.

A 64-element head-neck receiver RF coil (Siemens Healthineers) of which 30 elements were active was used. The cardiac rhythm was recorded with the integrated pulse oximeter device and used for the cardiac-resolved 4D reconstruction. A script for the extraction of the physiological signals from the raw MRI data is available at https://gitlab.com/chuv-tmrr/pmu-extraction.

To assess intra-session repeatability in the FR gradient-recalled echo sequence, a scan-rescan protocol was used for the subset of 14 participants previously described. Between acquisitions, the participant stood up briefly before being repositioned for the second scan. Brachial systolic and diastolic pressure measurements were acquired before and after the scans with a MR-compatible oscillometric semi-automatic sphygmomanometer (Expression, Invivo, a division of Philips Medical Systems) in a supine position while the volunteer was on the scanner table, and averaged afterwards.

### MR image reconstruction

A Linux workstation with a 64-core CPU (AMD Ryzen Threadripper Pro 5995WX; AMD), 994GB of RAM, and an NVIDIA RTX 6000 Ada Generation GPU (Nvidia) was used to perform the offline MRI reconstruction in MATLAB R2023b (The MathWorks). The reconstruction time ranged from 1 to 2 h.

Pulse oximetry was used to retrospectively order the continuously acquired FR k-space data into cardiac-motion-resolved bins. The cardiac triggers were used to sort the data in non-overlapping phases whose duration was optimized by comparing the estimated diameters at different bin widths (40, 80, 160, and 240 ms) with the corresponding diameters measured *via* ultrasound. The number of cardiac bins was dependent on the subject’s heart rate: the average heartbeat duration was computed and then divided by the bin width, resulting in the number of bins per cardiac cycle.

A k-t sparse sensitivity encoding‒SENSE compressed sensing algorithm [17, 18] was used to reconstruct 4D (3D + cardiac cycle) dynamic carotid images by solving an optimization problem:

1 $$\hat{x}={{{\rm{argmin}}}}_{{{\rm{x}}}}\left({{||}{{\bf{FC}}}{{\rm{x}}}-{{\rm{y}}}{||}}_{2}^{2}+{{{{\rm{\lambda }}}}_{{{\rm{c}}}}{{\rm{||}}}{\nabla }_{{{\rm{c}}}}{{\rm{x||}}}}_{1}\right)$$

where $\hat{x}$ is the reconstructed image, ***F*** and ***C*** are the non-uniform Fourier transform operator and the coil sensitivity, y is the k-space raw data, ∇_c_ is the first-order finite difference operator along the cardiac dimension and λ_c_ the optimized cardiac regularization weight. The optimization problem was solved using the Alternating Direction Method of Multipliers algorithm [22] with 10 iterations. The images were post-processed using high-dimensionality undersampled patch-based reconstruction (HD-PROST) [23] (Supplementary Fig. S1) to reduce residual noise as previously done [24, 25].

### Ultrasound image analysis

The M-mode ultrasound images were manually segmented by an expert neurologist and certified neurosonologist (T.B., with 2.5 years of experience) in a randomized order. Here, the upper and lower lumen-vessel wall boundaries were delineated during 5 heartbeats using a custom-written MATLAB application (Fig. 1c). The diameter evolution was automatically computed after the interpolation of the two delineated boundaries, their subtraction and linear detrending (Fig. 1e). The five systolic and diastolic peaks were determined using a multi-scale peak and trough detection [26] algorithm and averaged afterward (D_s_ and D_d_, respectively). The delineation was performed following the outer wall of the carotid arteries, and thus the D_s_ and D_d_ were corrected with the intima-media thickness value by subtracting it twice from both D_s_ and D_d_.

### MR image analysis

In the MR images, the carotid diameter was computed using a full-width at half-maximum (FWHM, Fig. 1d) approach [21, 27], defined as the width between two points corresponding to half the signal range. In an axial image, two regions of interest, which included the LC and RC, were manually selected. The lumen perimeter was then automatically delineated, and the carotid area A was calculated using a triangulation approach. The diameter of the carotid artery was estimated as D = 2$\sqrt{A/\pi }$ for all cardiac phases. D_s_ and D_d_ were defined as the maximum and minimum of the obtained curve (Fig. 1f). D_s_ and D_d_ were calculated for both the LC and RC and averaged for 3 slices, 1‒2 cm below the carotid bifurcation. No intima-media thickness correction was needed as the lumen signal was targeted directly.

### Carotid arterial stiffness

The carotid stiffness parameters were calculated considering the relation between the arterial diameter and the systemic blood pressure. The RDC, expressed as the percentage difference between D_s_ and D_d_ (Eq. 2), is an index of strain and thus of arterial deformation. β was then calculated to describe the stress–strain relationship based on the diameters and pressures (Eq. 3). Arterial compliance (AC), which reflects the ability of an artery to expand and contract in response to changes in pressure, was determined according to Eq. 4. Local pulse wave velocity (PWV) was estimated using a one-point approach [28] and β (Eq. 5), assuming a blood density (ρ) of 1050 kg/m³ at body temperature. The pressure–strain Young’s elastic modulus (E) was derived (Eq. 6) to evaluate the resistance of the artery to deformation when the pressure changes.

2 $${{\rm{RDC}}}=\frac{{{{\rm{D}}}}_{{{\rm{s}}}}-\,{{{\rm{D}}}}_{{{\rm{d}}}}}{{{{\rm{D}}}}_{{{\rm{d}}}}}100\,( \% )$$

3 $${{\rm{\beta }}}=\frac{{\mathrm{ln}}({{{\rm{P}}}}_{{{\rm{s}}}}/{{{\rm{P}}}}_{{{\rm{d}}}})}{({{{\rm{D}}}}_{{{\rm{s}}}}-{{{\rm{D}}}}_{{{\rm{d}}}})/{{{\rm{D}}}}_{{{\rm{d}}}}}$$

4 $${{\rm{AC}}}=\pi \frac{{{{{\rm{D}}}}_{{{\rm{s}}}}}^{2}-{{{{\rm{D}}}}_{{{\rm{d}}}}}^{2}}{4({{{\rm{P}}}}_{{{\rm{s}}}}-{{{\rm{P}}}}_{{{\rm{d}}}})}({{{\rm{mm}}}}^{2}/{{\rm{kPa}}})$$

5 $${{\rm{PWV}}}=\,\sqrt{{{\rm{\beta }}}\frac{{{{\rm{P}}}}_{{{\rm{s}}}}}{2{{\rm{\rho }}}}}({{\rm{m}}}/{{\rm{s}}})$$

6 $${{\rm{E}}}=\frac{({{{\rm{P}}}}_{{{\rm{s}}}}-{{{\rm{P}}}}_{{{\rm{d}}}})}{({{{\rm{D}}}}_{{{\rm{s}}}}-{{{\rm{D}}}}_{{{\rm{d}}}})/{{{\rm{D}}}}_{{{\rm{d}}}}}({{\rm{kPa}}})$$

All parameters were computed separately for the LC and RC arteries, as well as their average.

### Statistical analysis

The Shapiro–Wilk test was used to assess normality. Two-sided paired Student’s *t*-tests were used to compare normally distributed groups, and Wilcoxon signed-rank tests were used in case of non-normally distributed data. All results are reported as mean ± standard deviation for normal data or median (interquartile range) for non-normal data. A *p*-value lower than 0.05 was considered statistically significant. Repeatability of the metrics was quantified using the intraclass correlation coefficient (ICC) with a (2,1) formula [29] and the coefficient of variation (CV). This analysis was performed on diastolic and systolic diameter measurements from the LC and RC arteries in both modalities. Bland–Altman analyses (with the mean difference and the 95% limits of agreement 1.96 × standard deviation) and linear regressions (with Pearson correlation *r*) were also employed to evaluate the agreement and linear correlation, respectively, between modalities.

## Results

The optimal width of the cardiac bins was 80 ms, which resulted in the lowest mean squared error with respect to the ultrasound diameter measurements (Supplementary Fig. S2). The optimal cardiac regularization weight λ_c_ after reconstructing with varying regularization weights (Supplementary Fig. S3) was 0.01.

**Fig. 2:**
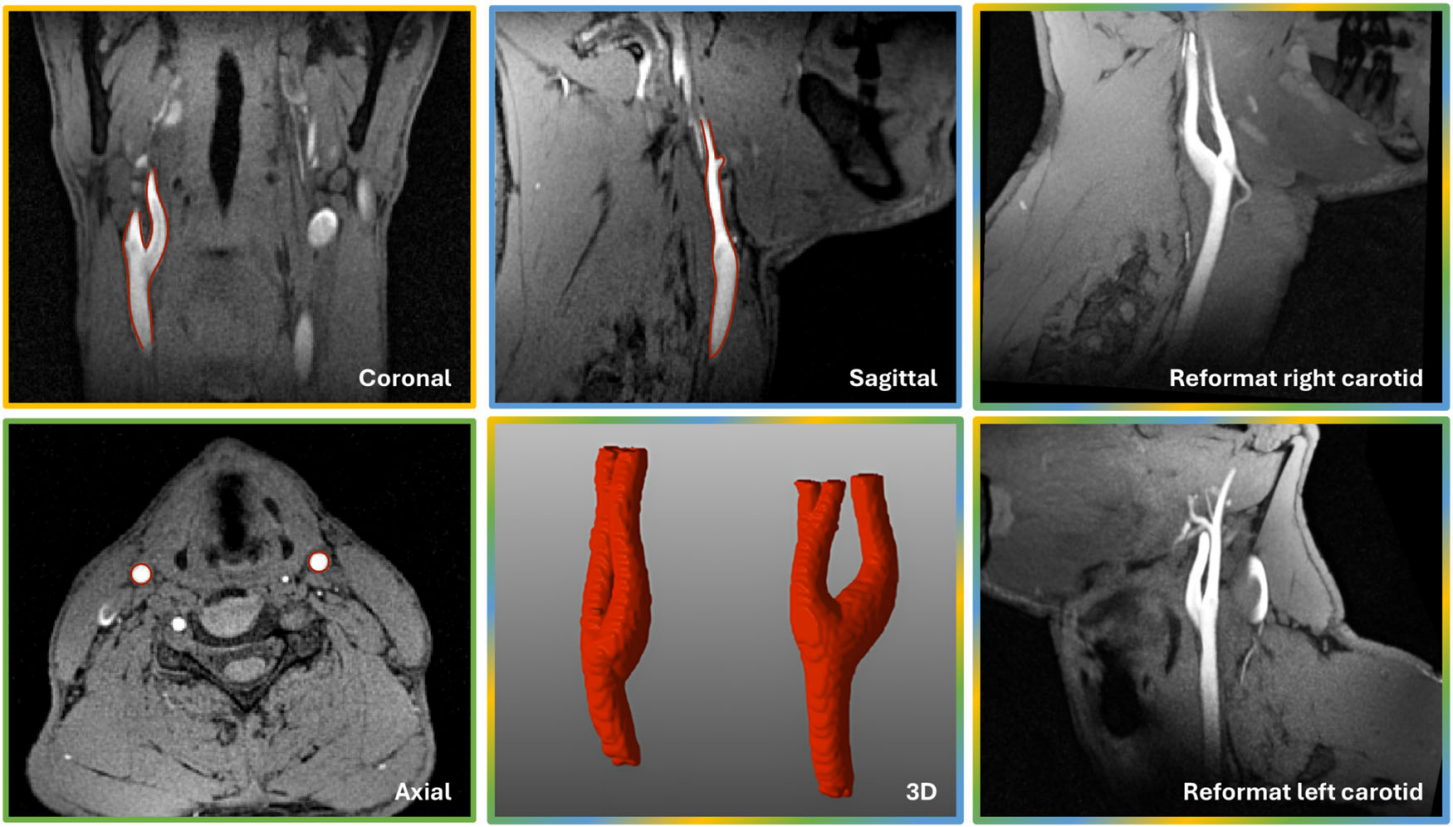
Three example orthogonal anatomical views of the neck and carotid arteries on magnetic resonance angiography (MRA) in a 42-year-old healthy male volunteer, together with a three-dimensional (3D) rendering and reformat of the carotid arteries. While the arterial blood has a bright signal supported by inflow, the lipid signals are largely suppressed by the water-excitation radiofrequency pulses

For all participants, the diameter measurement analysis showed an excellent repeatability for MRA (Fig. 2, Table 1) with high ICCs in both the LC (0.95 (0.65‒0.98)) and RC (0.93 (0.76‒0.97)). In ultrasound, the diameter measurements resulted in a relatively high ICC for the LC (0.78 (0.57‒0.89)) and a moderate ICC for the RC (0.51 (0.17‒0.74)). Consistently, the CVs were lower for both carotid arteries for MRA (CV_LC_ = 1.7%, CV_RC_ = 1.9%) compared to ultrasound (CV_LC_ = 3.8%, CV_RC_ = 4.6%).

**Table 1:** Interscan repeatability analysis

		ICC (95% CI)	CV
Magnetic resonance angiography	Right carotid	0.93 (0.76‒0.97)	1.9%
	Left carotid	0.95 (0.65‒0.98)	1.7%
Ultrasound	Right carotid	0.51 (0.17‒0.74)	4.6%
	Left carotid	0.78 (0.57‒0.89)	3.8%

*CI* Confidence interval, *CV* Coefficient of variation, *ICC* Intraclass coefficient

**Fig. 3:**
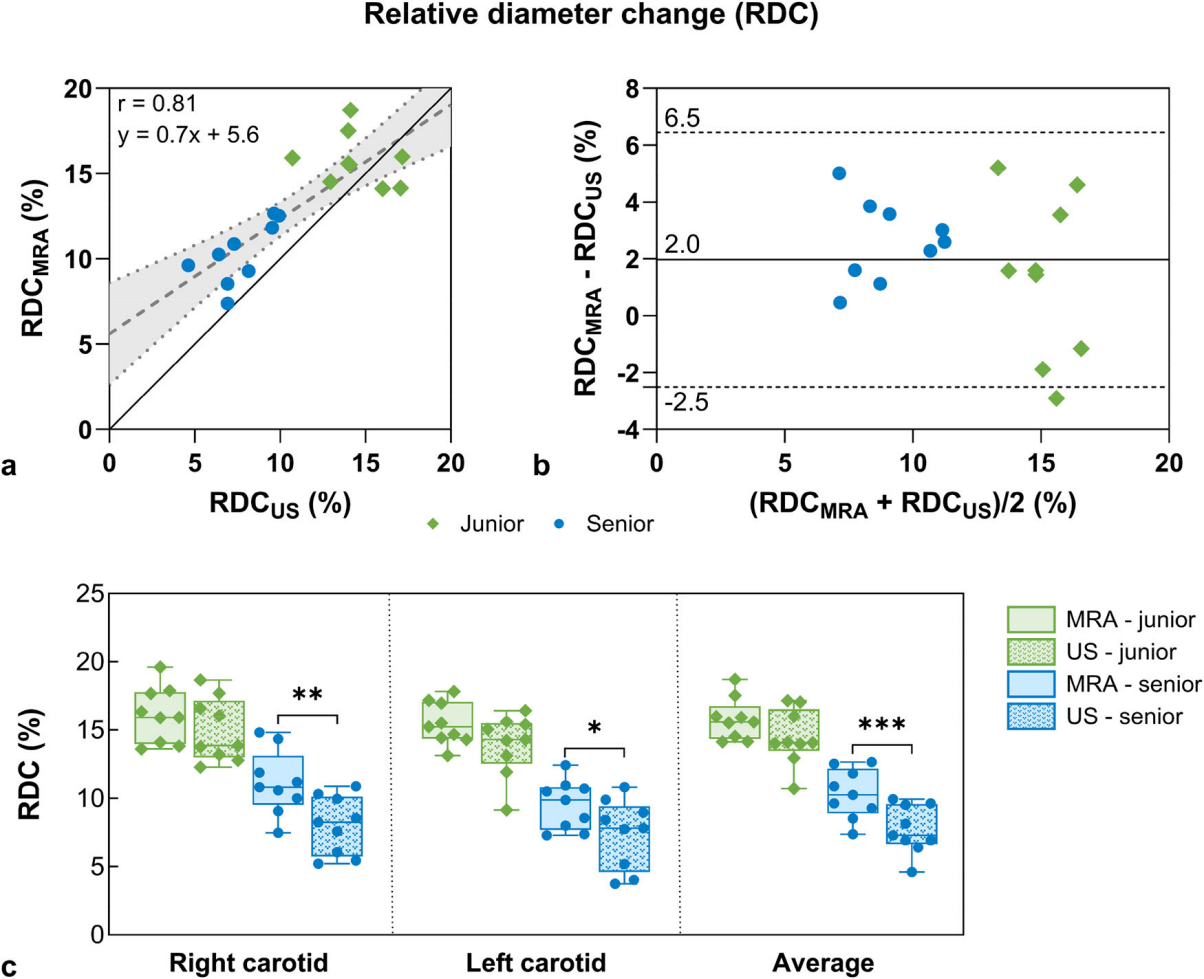
Comparison of magnetic resonance angiography (MRA)- and ultrasound (US)-derived relative diameter change (RDC). **a** Linear regression between ultrasound and MRA RDC with their linear fit (dashed line and equation) and junior (green diamonds) and senior (blue dots) cohorts represented. The continuous black line is the identity line (*y* = *x*). **b** Bland–Altman analysis of the RDC metric. **c** RDC box plots derived from MRA (solid bars) and ultrasound (dotted bars) for the right and left carotid arteries, as well as their average value. Statistical differences were observed between modalities for the senior cohort (*p*_RC_ = 0.001, *p*_LC_ = 0.038 and *p*_avg_ < 0.001)

The RDC linear regression (Fig. 3a) indicated a relatively strong positive correlation (r = 0.81) while Bland–Altman analyses (Fig. 3b, Supplementary Fig. S4) demonstrated a moderate bias and confidence intervals, and thus a moderate agreement between modalities. In both age cohorts, the RDC was higher when using MRA instead of ultrasound (RDC_junior_MRA_ = 15.8 ± 1.5% *versus* RDC_junior_ultrasound_ = 14.5 ± 2.0%, *p* = 0.369, Table 2), although it was only significant in the senior cohort (RDC_senior_MRA_ = 10.3 ± 1.8% *versus* RDC_senior_ultrasound_ = 7.7 ± 1.8%, *p* < 0.001, Fig. 3c). For both modalities, the RDC was significantly lower in the senior cohort compared to the junior cohort (*p* < 0.001).

**Table 2:** Comparison of the stiffness parameters for MRA and ultrasound, left and right carotid arteries and its average value, for both cohorts

		MRA			Ultrasound			*p*-value		
Parameter	Cohort	RC	LC	Average	RC	LC	Average	RC	LC	Average
P_d_ (mmHg)	Junior		63 ± 7			71 ± 6			**0.003**	
	Senior		71 ± 5			81 ± 8			**0.003**	
P_s_ (mmHg)	Junior		113 ± 8			117 ± 10			0.145	
	Senior		123 ± 14			134 ± 16			**0.007**	
D_d_ (mm)	Junior	5.5 ± 0.4	5.6 ± 0.6	5.5 ± 0.5	5.3 ± 0.5	5.6 ± 0.6	5.5 ± 0.5	0.406	0.940	0.547
	Senior	5.9 ± 0.3	6.0 ± 0.4	6.0 ± 0.3	5.5 ± 0.3	5.8 ± 0.5	5.7 ± 0.4	**0.013**	0.134	**0.032**
D_s_ (mm)	Junior	6.4 ± 0.4	6.4 ± 0.6	6.4 ± 0.5	6.1 ± 0.5	6.4 ± 0.7	6.2 ± 0.6	0.155	0.368	0.140
	Senior	6.6 ± 0.4	6.6 ± 0.4	6.6 ± 0.4	6.0 ± 0.4	6.2 ± 0.6	6.1 ± 0.5	**0.003**	**0.009**	**0.003**
RDC (%)	Junior	16.1 ± 2.0	15.5 ± 1.5	15.8 ± 1.5	15.0 ± 2.3	13.9 ± 2.2	14.5 ± 2.0	0.369	0.123	0.198
	Senior	11.1 ± 2.3	9.5 ± 1.8	10.3 ± 1.8	8.0 ± 2.1	7.4 ± 2.5	7.7 ± 1.8	**0.001**	**0.038**	**< 0.001**
β (-)	Junior	3.7 ± 1.0	3.8 ± 0.8	3.8 ± 0.9	3.4 ± 0.7	3.7 ± 0.9	3.5 ± 0.7	0.384	0.712	0.519
	Senior	5.0 ± 1.2	6.0 (1.7)	5.4 ± 1.0	6.5 ± 1.7	6.0 (6)	7.2 ± 3.0	**0.022**	0.477	0.082
AC (mm^2^/kPa)	Junior	1.26 ± 0.21	1.24 ± 0.20	1.25 ± 0.19	1.20 ± 0.26	1.22 ± 0.29	1.21 ± 0.26	0.380	0.820	0.575
	Senior	0.97 ± 0.24	0.86 ± 0.28	0.92 ± 0.23	0.60 ± 0.23	0.64 ± 0.31	0.62 ± 0.25	**0.002**	0.082	**0.010**
PWV (m/s)	Junior	5.1 ± 0.8	5.2 ± 0.6	5.2 ± 0.7	5.0 ± 0.6	5.1 ± 0.7	5.1 ± 0.6	0.615	0.944	0.770
	Senior	6.2 ± 1.0	6.7 ± 0.9	6.5 ± 0.9	7.4 ± 1.2	8.1 ± 2.9	7.7 ± 1.9	**0.014**	0.158	0.051
E (kPa)	Junior	42.1 ± 11.5	43.3 ± 9.3	42.7 ± 10.1	41.3 ± 7.8	44.8 ± 10.9	43.0 ± 8.7	0.829	0.746	0.940
	Senior	64.1 ± 19.6	76.4 (31)	68.8 ± 17.5	91.0 ± 26.3	81.5 (86.2)	103.7 ± 50.3	**0.008**	0.286	**0.042**

Values are represented as mean ± standard deviation for normal data or median (interquartile range) for non-normal data. Junior (*n* = 9), Senior (*n* = 9). *p*-values in bold are statistically significant.

*AC* Arterial compliance, *D*_*d*_ Diastolic diameter, *D*_*s*_ Systolic diameter, *E* Pressure–strain Young’s elastic modulus, *LC* Left carotid, *MRA* Magnetic resonance angiography, *P*_*d*_ Diastolic brachial pressure, *P*_*s*_ Systolic brachial pressure, *PWV* Local pulse wave velocity, *RC* Right carotid, *RDC* Relative diameter change, *β* Stiffness index

**Fig. 4:**
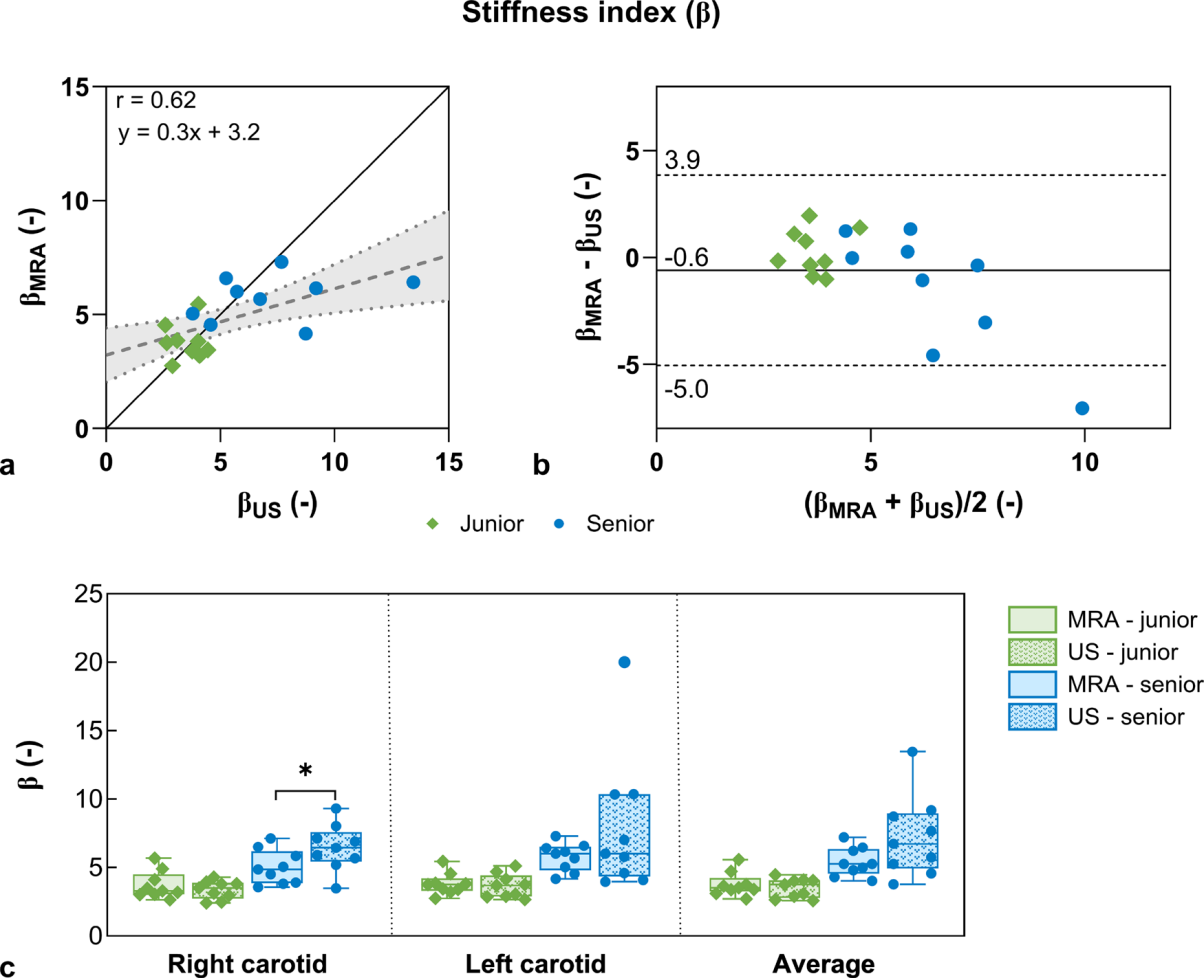
Comparison of magnetic resonance angiography (MRA)- and ultrasound (US)-derived carotid stiffness index (β). **a** Linear regression between ultrasound and MRA β metrics. The dashed blue line shows the linear fit of the data. The continuous black line refers to *y* = *x*. Green diamonds represent the junior cohort, while blue dots represent the senior cohort. The linear fit and Pearson correlation coefficient (r) are displayed. **b** Bland–Altman analysis of ultrasound and MRA β metrics. **c** β box plots derived from MRA (solid bars) and ultrasound (dotted bars) for the right and left carotid arteries, as well as their average value. Statistical differences were observed between modalities for the senior cohort (blue bars) in the right carotid (*p*_RC_ = 0.022)

The linear regression of β between the two modalities (Fig. 4a) indicated moderate correlation between MRA and ultrasound (*r* = 0.62), while Bland–Altman analysis (Fig. 4b) showed good agreement with a bias of -0.6. ultrasound had larger intersubject variability in the senior group, although there was no significant β difference for the junior (*p* = 0.519) or senior (*p* = 0.082) cohorts (Fig. 4c). When comparing cohorts, the β measured with MRA (β_senior_MRA_ = 5.4 ± 1.0 *versus* β_junior_MRA_ = 3.8 ± 0.9, *p* = 0.002) and ultrasound (β_senior_ ultrasound_ = 7.2 ± 3.0 *versus* β_junior_ ultrasound_ = 3.5 ± 0.7, *p* = 0.005) was significantly higher in the senior group.

**Fig. 5:**
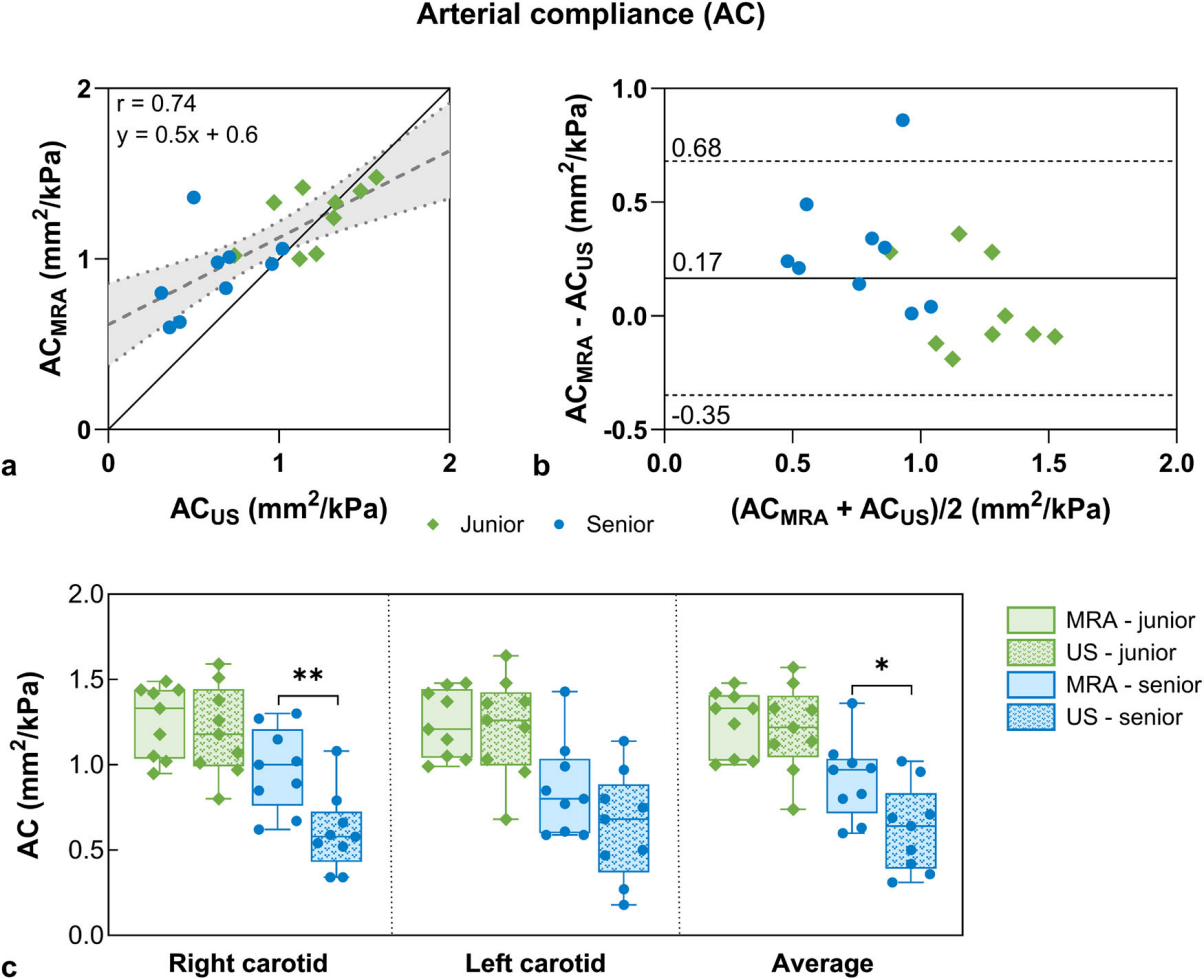
Comparison of magnetic resonance angiography (MRA)- and ultrasound (US)-derived arterial compliance (AC). **a** Linear regression between ultrasound and MRA AC metrics with their linear fit (dashed line) and junior (green diamonds) and senior (blue dots) cohorts represented. The continuous black line refers to *y* = *x*. The linear fit and Pearson correlation coefficient (r) are displayed. **b** Bland–Altman analysis of ultrasound and MRA AC metrics. **c** AC box plots derived from MRA (solid bars) and ultrasound (dotted bars) for the right and left carotid arteries, as well as their average value. Statistical differences were observed between modalities for the senior (blue dots) cohort (*p*_senior_RC_ = 0.002 and *p*_senior_avg_ = 0.010)

The AC linear regression indicated a strong correlation (*r* = 0.74, Fig. 5a), while there was a small AC bias of 0.2 mm^2^/kPa between the two modalities (Fig. 5b). The AC measured with MRA was higher than with ultrasound for both the junior (AC_junior_MRA_ = 1.3 ± 0.2 mm^2^/kPa *versus* AC_junior_ultrasound_ = 1.2 ± 0.3 mm^2^/kPa, *p* = 0.575) and senior cohorts (AC_senior_MRA_ = 0.9 ± 0.2 mm^2^/kPa *versus* AC_senior_ultrasound_ = 0.6 ± 0.3 mm^2^/kPa, *p* = 0.010, Fig. 5c). The AC was significantly lower for the senior cohort with respect to the junior cohort for both MRA (*p* = 0.004) and ultrasound (*p* < 0.001).

**Fig. 6:**
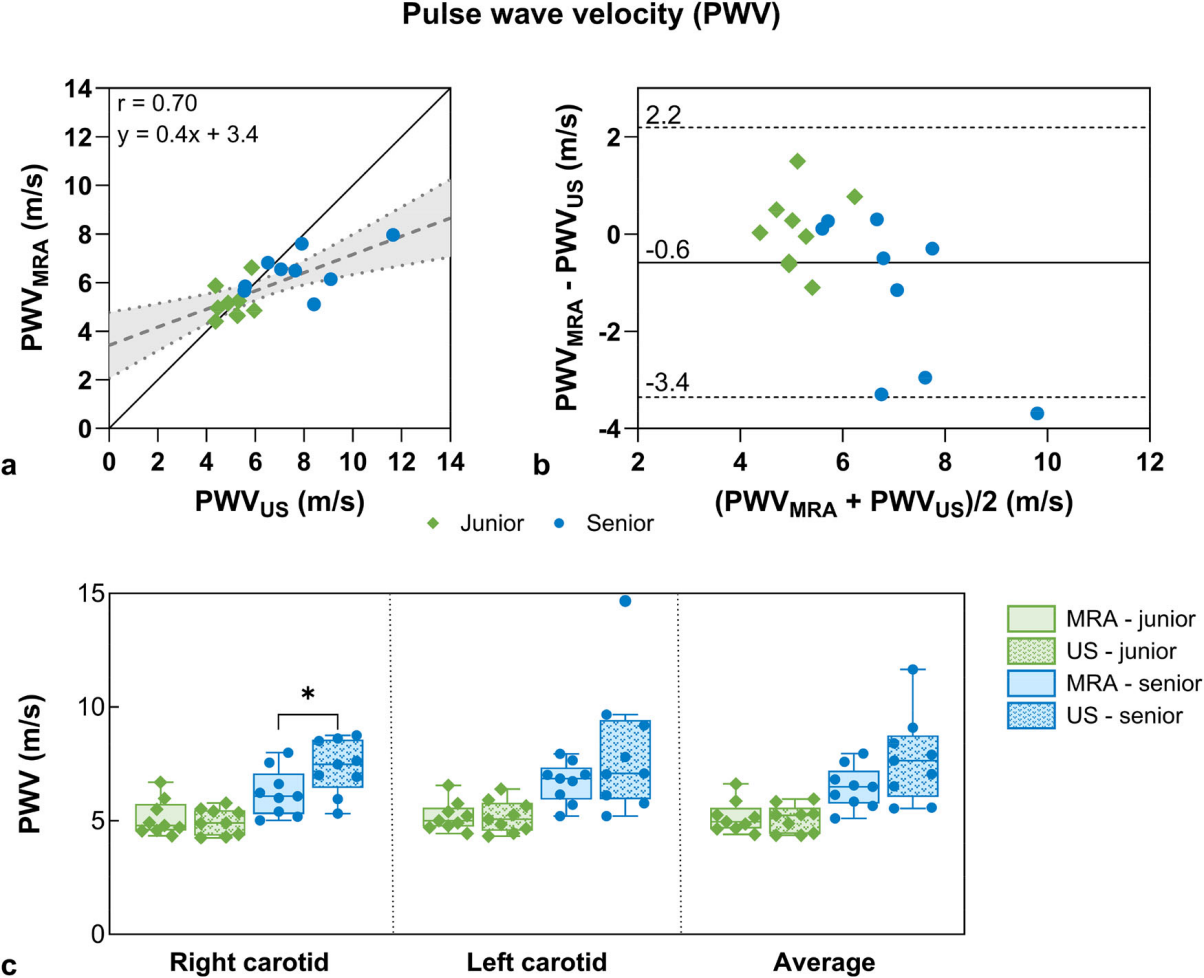
Comparison of magnetic resonance angiography (MRA)- and ultrasound (US)-derived local pulse wave velocity (PWV). **a** Linear regression between ultrasound and MRA PWV metrics with their linear fit (dashed line) and junior (green diamonds) and senior (blue dots) cohorts represented. The continuous black line refers to *y* = *x*. The linear fit and Pearson correlation coefficient (r) are displayed. **b** Bland–Altman analysis of ultrasound and MRA PWV metrics with the mean difference and the 95% limits of agreement (1.96 standard deviation). **c** PWV box plots derived from MRA (solid bars) and ultrasound (dotted bars) for the right and left carotid arteries, as well as their average value. Statistical differences were observed between modalities for the senior cohort in the right carotid (*p*_RC_ = 0.014)

For PWV, strong correlation (*r* = 0.70, Fig. 6a) and good agreement with a bias of -0.6 m/s and confidence intervals ranging from -3.4 to 2.2 m/s were observed (Fig. 6b). For the all-participant PWV there were no significant differences between the two modalities (Fig. 6c). The PWV was significantly higher in the senior cohort for both MRA (PWV_junior_MRA_ = 5.2 ± 0.7 m/s *versus* PWV_senior_MRA_ = 6.5 ± 0.9 m/s, *p* < 0.001) and ultrasound (PWV_junior_ultrasound_ = 5.1 ± 0.6 m/s *versus* PWV_senior_ultrasound_ = 7.7 ± 1.9 m/s, *p* = 0.003).

**Fig. 7:**
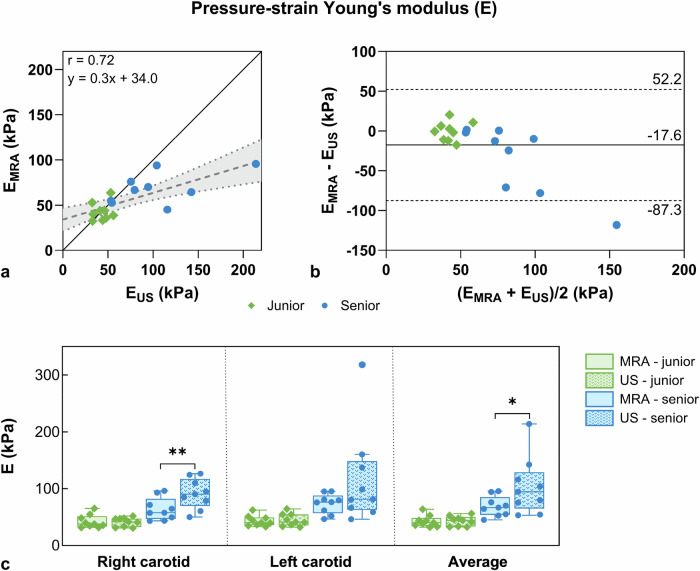
Comparison of magnetic resonance angiography (MRA)- and ultrasound (US)-derived pressure–strain Young’s modulus (E). **a** Linear regression between ultrasound and MRA E metrics with their linear fit (dashed line). The continuous black line refers to *y* = *x*. The linear fit and Pearson correlation coefficient (*r*) are displayed and junior (green diamonds) and senior (blue dots) cohorts are represented. **b** Bland–Altman analysis of ultrasound and MRA E metrics with the mean difference and the 95% limits of agreement (1.96 standard deviation). **c** E box plots derived from MRA (solid bars) and ultrasound (dotted bars) for the right and left carotid arteries, as well as their average value. Statistical differences were observed between modalities for the senior cohort in the right carotid (*p*_RC_ = 0.008) and in the average of both carotids (*p*_avg_ = 0.042)

Finally, the linear regression plot for E (Fig. 7a) showed strong agreement between modalities (r = 0.72). Bland–Altman analysis (Fig. 7b) showed a bias of -17.6 kPa with broad confidence intervals ranging from -87.3 to 52.2 kPa. When comparing both modalities, there was a significant difference in the senior cohort (*p* = 0.042, Fig. 7c), whereas no significant difference was observed for the junior cohort. The MRA E-modulus for the senior cohort (E_senior_MRA_ = 68.8 ± 17.5 kPa) was significantly higher than that of the junior cohort (E_junior_MRA_ = 42.7 ± 10.1 kPa, *p* = 0.002). Similarly, the ultrasound E-modulus was higher in the senior cohort (E_senior_ultrasound_ = 103.7 ± 50.3 kPa) compared to the junior cohort (E_junior_ultrasound_ = 43.0 ± 8.7k Pa, *p* = 0.007).

## Discussion

Dynamic FR carotid MRA with slab-selective water-excitation RF pulses was successfully acquired in all participants. It was used to repeatably assess the diameter differences between the diastolic and systolic phases and derive several stiffness-related parameters. To account for possible discrepancies due to different temporal resolutions between modalities, the cardiac bin width was optimized for the MRA reconstruction. This width was chosen as a compromise between the reconstruction time and the mean squared error compared to ultrasound measurements. The comparable, and occasionally larger, diameter changes detected by MRI relative to ultrasound suggest that the chosen temporal resolution was sufficient to capture systolic and diastolic peak dynamics.

Repeatability was higher in MRA than in ultrasound, as indicated by a higher ICC and lower CV. This could be due to both image quality differences, operator involvement in ultrasound, and manual *versus* semi-automatic analysis. In comparison to the semi-automatic segmentation of the carotid lumen in MRA, we used a manual segmentation technique for the delineation of the inner carotid vessel wall in ultrasound, which can also affect repeatability. However, the MR images lent themselves well for semi-automated analysis while the ultrasound images did not, probably contributing to the higher repeatability of MRA: the semi-automated segmentation of a bright circular shape was straightforward to implement, while such automation for the more complex pattern seen in ultrasound would likely require large datasets and deep learning methods, which are not feasible with a dataset of this size.

Several groups have studied the relation of carotid arterial stiffness with the development of cardiovascular disease and stroke using ultrasound [5–7, 30]. In terms of age-related changes, it was found that parameters such as β, PWV, E and diastolic and systolic diameters increased with age due to changes in the vessel wall structure and arterial remodeling, while AC decreased [12, 13, 31–33]. Increases in the PWV also indicated a higher risk of the development of cardiovascular disease [34]. Therefore, carotid arterial stiffness parameters can be a predictor of the occurrence of cardiovascular disease and stroke in different patient populations. However, the routine ultrasound that is used to obtain these metrics lacks the isotropic 3D visualization offered by MRA. While ultrasound is widely accessible, it requires skilled operators and a standardized head-neck rotation to examine the upper branches of the carotid artery. In addition, imaging of the more distal segments is often limited by anatomical constraints, such as interference from the maxillofacial bones. Finally, while operators aim to use minimal contact pressure between the probe and the skin, even low pressure may affect the diastolic diameter measurement (and thus the derived metrics).

When comparing the age cohorts, we observed significant differences for all parameters in both modalities. In the senior cohort, the RDC and the AC were lower than in the junior cohort. Conversely, β, PWV and E-modulus were higher, which agrees well with the aforementioned previous studies [12, 13, 31–33]. Although our study included relatively small subgroups, the observed effect sizes were large to very large (Cohen d = 1.56–1.81), corresponding to ~88–95% power for detecting age-related differences at a two-sided α = 0.05. While the study was not powered to detect smaller effects, it was sufficient to identify the large age-related changes. Overall, there was a relatively good agreement and correlation between modalities. The intermodality confidence intervals that we obtained for β, PWV and AC were similar to those reported for intra- and interobserver variability for ultrasound [10]. Besides AC, the stiffness parameters estimated for the junior cohort did not show any significant differences between modalities. In contrast, the senior cohort showed differences in RDC, AC and E. These discrepancies may be attributed to small variations in diameter estimations between modalities, which have previously been shown to significantly influence the calculation of stiffness-related indices [3]. On the one hand, during systole, blood velocity increases [35], which in MRA leads to more unsaturated blood magnetization and thus a higher signal intensity throughout the entire lumen of the vessel. On the other hand, due to the decreased flow during diastole, there may be a relative accumulation of saturated blood near the vessel wall, causing a reduction in signal intensity compared to the center of the vessel in MRA. The two effects together may lead to an RDC overestimation due to inflow-related signal changes, which could be mitigated by performing a scout scan to measure flow rates and thereby determine the appropriate TR that minimizes blood saturation while maintaining a reasonable acquisition time. Conversely, the image quality in several ultrasound images was suboptimal (but still well within diagnostic limits), which may also have made the correct delineation of the lumen-vessel wall boundaries more challenging. All these factors could affect the estimation of the diastolic and systolic diameters. The diameters and β measured with MRA were in line with those measured in previous studies with different MRI techniques [36–38]. PWV can also be derived from 4D flow sequences, which provide several additional flow-related metrics but no dynamic vessel diameter information due to limited contrast between the blood and vessel wall [39]. Nevertheless, these studies computed only one parameter for arterial stiffness (*i.e*., diameter changes, distensibility coefficients or pulse wave velocities), while we quantified the four main indices used to assess carotid arterial stiffness in a clinical setting, without the need for contrast agents and in a relatively short time.

Although only a single location of the common carotid artery was analyzed in this study, as it was inherent to the 1D ultrasound comparison, our 3D MRA protocol has an isotropic 0.6 mm resolution. This should enable assessment of the vessel stiffness along the internal and external carotid arteries from the same scans, as well as geometrical properties such as the bifurcation angle [40]. Similarly, it should be feasible to map regional stiffness metrics onto the vessel wall for a comprehensive 3D visualization of the stiffness heterogeneity, which represents an advantage over 2D ultrasound. In contrast, 2D cine protocols are typically optimized for lower in-plane spatial resolutions [41], while their thick slice dimension precludes precise 3D measurements. However, 3D assessment requires an adequate segmentation of the vessel lumen for all slices and all cardiac phases, which is time-consuming. Techniques such as B-spline snakes [42] or deep learning approaches [43] have already been successfully applied for the segmentation of the carotid artery in multi-contrast MRI to reduce analysis time from manual and semi-automatic techniques. Although necessary for clinical translation, its implementation was beyond the scope of this study.

Besides carotid pulsation, swallowing and respiration are also confounders in carotid MRA [44]. These motion sources might affect the estimation of the diameters due to unaccounted vessel displacements. Several efforts have been made to account for displacements when swallowing in a turbo spin echo sequence with a Cartesian k-space trajectory, showing an improvement in image sharpness [45]. Although not reported in this study, we also conducted preliminary tests to assess whether swallowing would affect image quality by having volunteers forcefully swallow multiple times, and in- or excluding that data during the reconstruction; we did not observe any visual differences in our image quality or quantitative differences in RDC. Cartesian k-space trajectories are known to be more sensitive to motion artifacts compared to radial trajectories [46, 47], which may explain the greater improvement in image quality observed in studies employing Cartesian sampling when motion correction is applied. Respiratory-corrected reconstructions are well-established in cardiac MRI, and using approaches such as focused navigation [48] for carotid imaging might improve image sharpness and enhance image quality.

This study had several limitations. First, this is a proof-of-concept study on healthy volunteers and did not include patients with significant carotid stenoses. Second, instead of in the carotid artery, pressure was measured as a surrogate in the brachial artery, which tends to be slightly higher [49], although this offset appears to have a limited impact on the derived stiffness parameters [13]. Third, we used M-mode ultrasound which is known to be less precise than echo-tracking [50] or speckle-tracking [51], potentially increasing variability in diameter estimations and intermodality comparisons in the senior cohort when image quality is suboptimal (*e.g*., due to increased subcutaneous fat tissue), although M-mode ultrasound is the clinical routine and reference standard that is available in most centers. In addition, while it matched the ultrasound measurements very well and was previously tested for coronary imaging [27], the FWHM-based segmentation approach was not validated against flow phantom measurements as a well-controlled reference standard, which could provide valuable insights into how this approach reflects true vessel diameter measurements. At present, the image reconstruction time prevents direct diagnostic use of the technique on the scanner. This limitation could be overcome by using deep learning–based reconstruction methods [52], which can reduce reconstruction time to minutes or even seconds. Finally, our study only included a relatively small number of participants without known cardiovascular disease, which limited the ability to detect small differences. Future work should therefore focus on using the MRA sequence, ideally in combination with an MRI protocol including vessel wall imaging, in patients with different degrees of carotid atherosclerotic plaque to investigate the relationship between carotid arterial stiffness and disease burden, as well as to explore its association with complementary stiffness measures such as central aortic stiffness.

In conclusion, we demonstrated the feasibility of characterizing carotid diameter changes and stiffness-related parameters using FR 4D dynamic MRA in two age groups. In comparison to routine 2D ultrasound assessments of carotid arterial stiffness, MRA showed an excellent scan-rescan repeatability. Combining 3D MRA with measures of carotid artery diameter across the cardiac cycle and derived stiffness-related parameters would enable a comprehensive evaluation of 3D arterial stiffness and morphology of the carotid artery. After appropriate validation in patients, this approach might provide a more comprehensive insight into a heterogeneous disease and could better facilitate diagnosis and inform therapy in carotid artery disease.

## Supplementary information

**Additional file 1**: **Fig. S1** —Calculation of the apparent signal-to-noise ratio (aSNR) before and after applying HD-PROST. The aSNR is calculated as the mean intensity of the carotid lumen divided by the standard deviation of the intensity in the sternocleidomastoid muscle as background noise between the non-denoised and denoised images made direct background measurements non consistent. **(a)** There is a significant increase (*p* < 0.0001) in aSNR when applying HD-PROST after compressed sensing (CS). **(b)** This improvement is also observed in axial images for in the junior (top row) and senior (bottom row), shown before (orange) and after (teal) HD-PROST. *HD-PROST* High-dimensionality undersampled patch-based reconstruction. **Fig. S2** —Evaluation of the diastolic and systolic diameters for the left (purple) and right (yellow) carotid arteries when using different bin widths (40, 80, 160 and 240 ms) for the magnetic resonance angiography (MRA) reconstruction. The mean squared error (MSE) was calculated by comparing the estimated diameters at each bin width with the corresponding diameters measured *via* ultrasound. It can be visually observed that the most significant variations occur in the diastolic phase, whereas the systolic phase remains relatively consistent across all bin widths. Increasing the bin width results in more averaged motion states, leading in an overestimation of the diameters in the diastolic phase. A bin width of 80 ms over 40 ms bin was selected for further analysis, as it yielded a low MSE while maintaining a reasonable reconstruction time. **Fig. S3** —Evaluation of mean squared error (MSE) between ultrasound- and magnetic resonance angiography (MRA)-derived parameters across varying cardiac regularization weights (λ_c_) for the right (yellow dots) and left (purple triangles) carotid arteries. Apparent signal-to-noise ratio (aSNR) was also calculated for each corresponding MRA image to assess image quality as the mean intensity inside the carotid over the standard deviation of the background noise. Overall, there is a lower MSE when using λ_c_ = 0.01. As expected, images reconstructed with λ_c_ = 0.05 presented a higher image quality: the higher the regularization, the smoother the resulting image. As most of the knee points coincide with the cardiac regularization weight λ_c_ = 0.01, this parameter was used for the reconstruction of all the images of the study. **Fig. S4** —Subgroup Bland–Altman plots for the stiffness parameters. In general, narrower confidence intervals and lower biases are observed for the junior cohort (green diamonds).

## Data Availability

The data supporting the findings of this study are not publicly available due to privacy and ethical restrictions, but are available from the corresponding author upon reasonable request.

## Abbreviations

1D: One-dimensional
2D: Two-dimensional
3D: Three-dimensional
4D: Four-dimensional
5D: Five-dimensional
AC: Arterial compliance
CV: Coefficient of variation
D: Diameter
D_d_: Diastolic diameter
D_s_: Systolic diameter
E: Pressure–strain Young’s elastic modulus
FR: Free-running
ICC: Intraclass correlation coefficient
LC: Left carotid
MR: Magnetic resonance
MRA: Magnetic resonance angiography
MRI: Magnetic resonance imaging
PWV: Pulse wave velocity
RC: Right carotid
RDC: Relative diameter change
RF: Radiofrequency
β: Stiffness index
