# Supplementary material for: Monitoring carotid arterial stiffness using non-contrast-enhanced 4D MR angiography
Source: Eur Radiol Exp. 2026 May 6;10:58. doi: 10.1186/s41747-026-00716-z (PMC13149800; doi:10.1186/s41747-026-00716-z)
Supplement: Supplementary file 1 — Additional file 1: Fig. S1 —Calculation of the apparent signal-to-noise ratio (aSNR) before and after applying HD-PROST. The aSNR is calculated as the mean intensity of the carotid lumen divided by the standard deviation of the intensity in the sternocleidomastoid muscle as background noise between the non-denoised and denoised images made direct background measurements non consistent. (a) There is a significant increase (p < 0.0001) in aSNR when applying HD-PROST after compressed sensing (CS). (b) This improvement is also observed in axial images for in the junior (top row) and senior (bottom row), shown before (orange) and after (teal) HD-PROST. HD-PROST High-dimensionality undersampled patch-based reconstruction. Fig. S2 —Evaluation of the diastolic and systolic diameters for the left (purple) and right (yellow) carotid arteries when using different bin widths (40, 80, 160 and 240 ms) for the magnetic resonance angiography (MRA) reconstruction. The mean squared error (MSE) was calculated by comparing the estimated diameters at each bin width with the corresponding diameters measured via ultrasound. It can be visually observed that the most significant variations occur in the diastolic phase, whereas the systolic phase remains relatively consistent across all bin widths. Increasing the bin width results in more averaged motion states, leading in an overestimation of the diameters in the diastolic phase. A bin width of 80 ms over 40 ms bin was selected for further analysis, as it yielded a low MSE while maintaining a reasonable reconstruction time. Fig. S3 —Evaluation of mean squared error (MSE) between ultrasound- and magnetic resonance angiography (MRA)-derived parameters across varying cardiac regularization weights (λc) for the right (yellow dots) and left (purple triangles) carotid arteries. Apparent signal-to-noise ratio (aSNR) was also calculated for each corresponding MRA image to assess image quality as the mean intensity inside the carotid over the standa [file 41747_2026_716_MOESM1_ESM.pdf]

Monitoring carotid arterial stiffness using non-contrast-enhanced 4D MR angiography

ELECTRONIC SUPPLEMENTARY MATERIAL

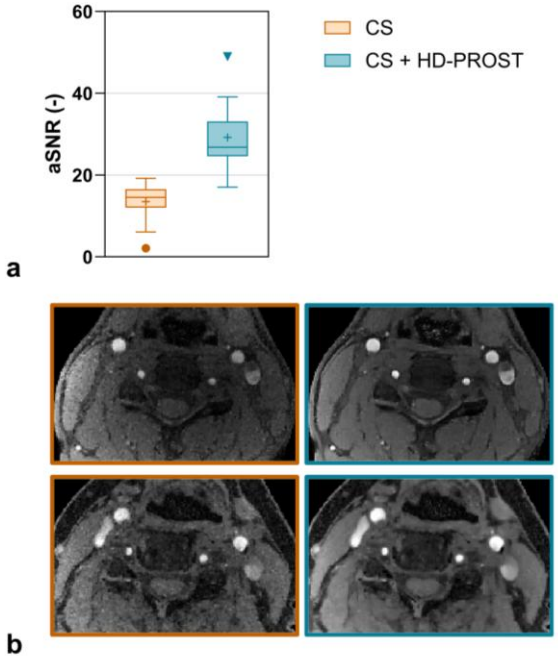

Fig. S1 —Calculation of the apparent signal-to-noise ratio (aSNR) before and after applying HD-PROST. The aSNR is calculated as the mean intensity of the carotid lumen divided by the standard deviation of the intensity in the sternocleidomastoid muscle as background noise between the non-denoised and denoised images made direct background measurements non consistent. **(a)** There is a significant increase ($p < 0.0001$) in aSNR when applying HD-PROST after compressed sensing (CS). **(b)** This improvement is also observed in axial images for in the junior (top row) and senior (bottom row), shown before (orange) and after (teal) HD-PROST. *HD-PROST* High-dimensionality undersampled patch-based reconstruction.

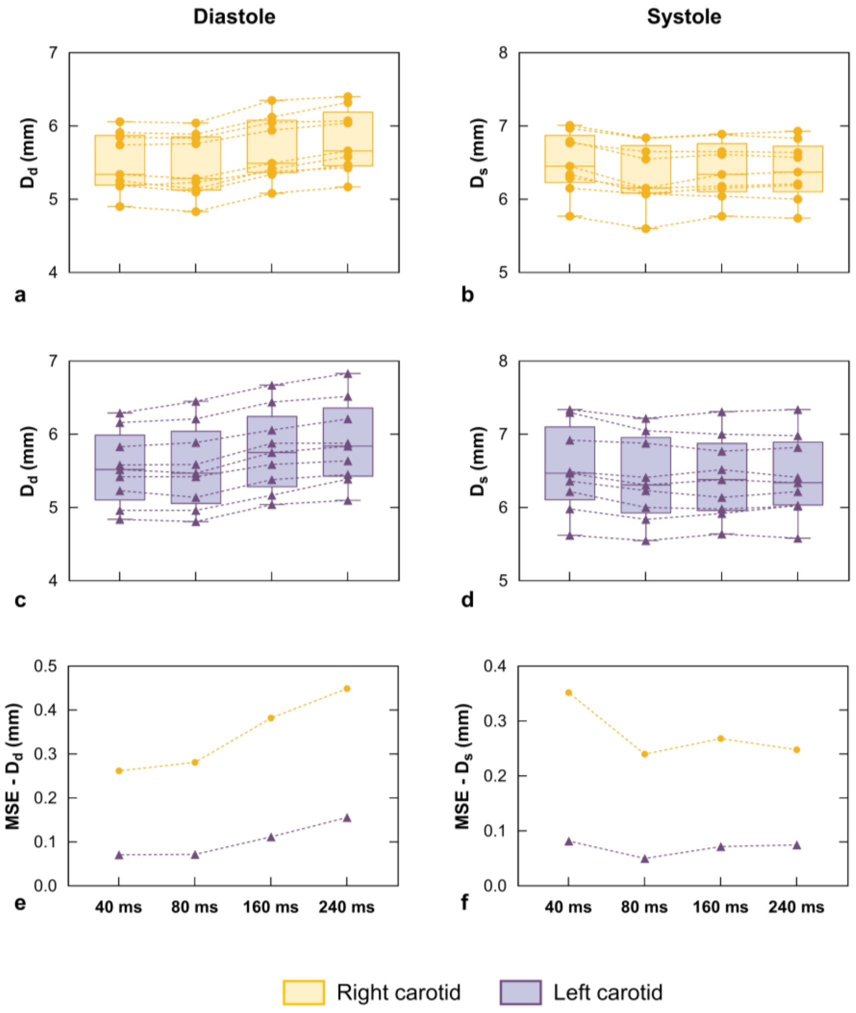

Fig. S2 —Evaluation of the diastolic and systolic diameters for the left (purple) and right (yellow) carotid arteries when using different bin widths (40, 80, 160 and 240 ms) for the magnetic resonance angiography (MRA) reconstruction. The mean squared error (MSE) was calculated by comparing the estimated diameters at each bin width with the corresponding diameters measured via ultrasound. It can be visually observed that the most significant variations occur in the diastolic phase, whereas the systolic phase remains relatively consistent across all bin widths. Increasing the bin width results in more averaged motion states, leading in an overestimation of the diameters in the diastolic phase. A bin width of 80 ms over 40 ms bin was selected for further analysis, as it yielded a low MSE while maintaining a reasonable reconstruction time.

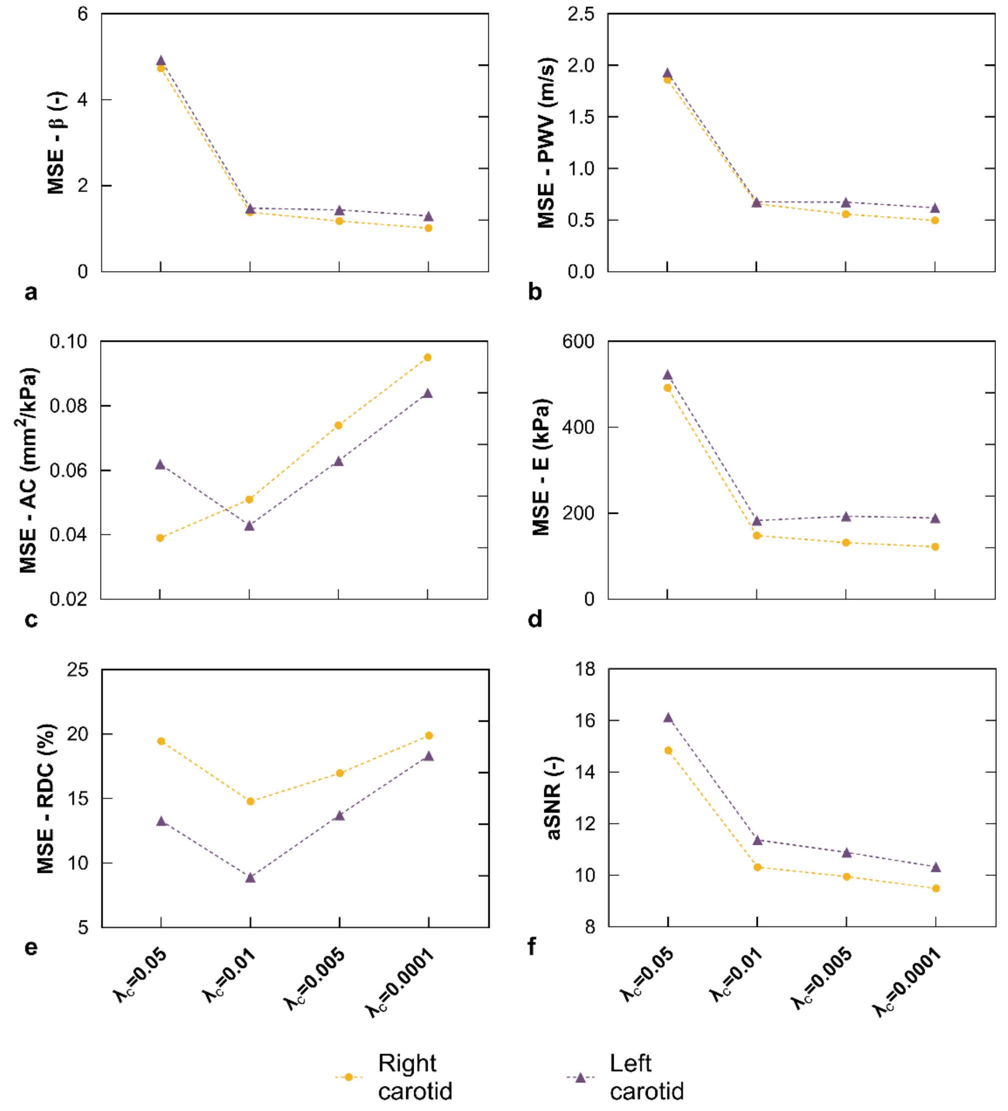

Fig. S3 —Evaluation of mean squared error (MSE) between ultrasound- and magnetic resonance angiography (MRA)-derived parameters across varying cardiac regularization weights (λ_c) for the right (yellow dots) and left (purple triangles) carotid arteries. Apparent signal-to-noise ratio (aSNR) was also calculated for each corresponding MRA image to assess image quality as the mean intensity inside the carotid over the standard deviation of the background noise. Overall, there is a lower MSE when using λ_c = 0.01. As expected, images reconstructed with λ_c = 0.05 presented a higher image quality: the higher the regularization, the smoother the resulting image. As most of the knee points coincide with the cardiac regularization weight λ_c = 0.01, this parameter was used for the reconstruction of all the images of the study.

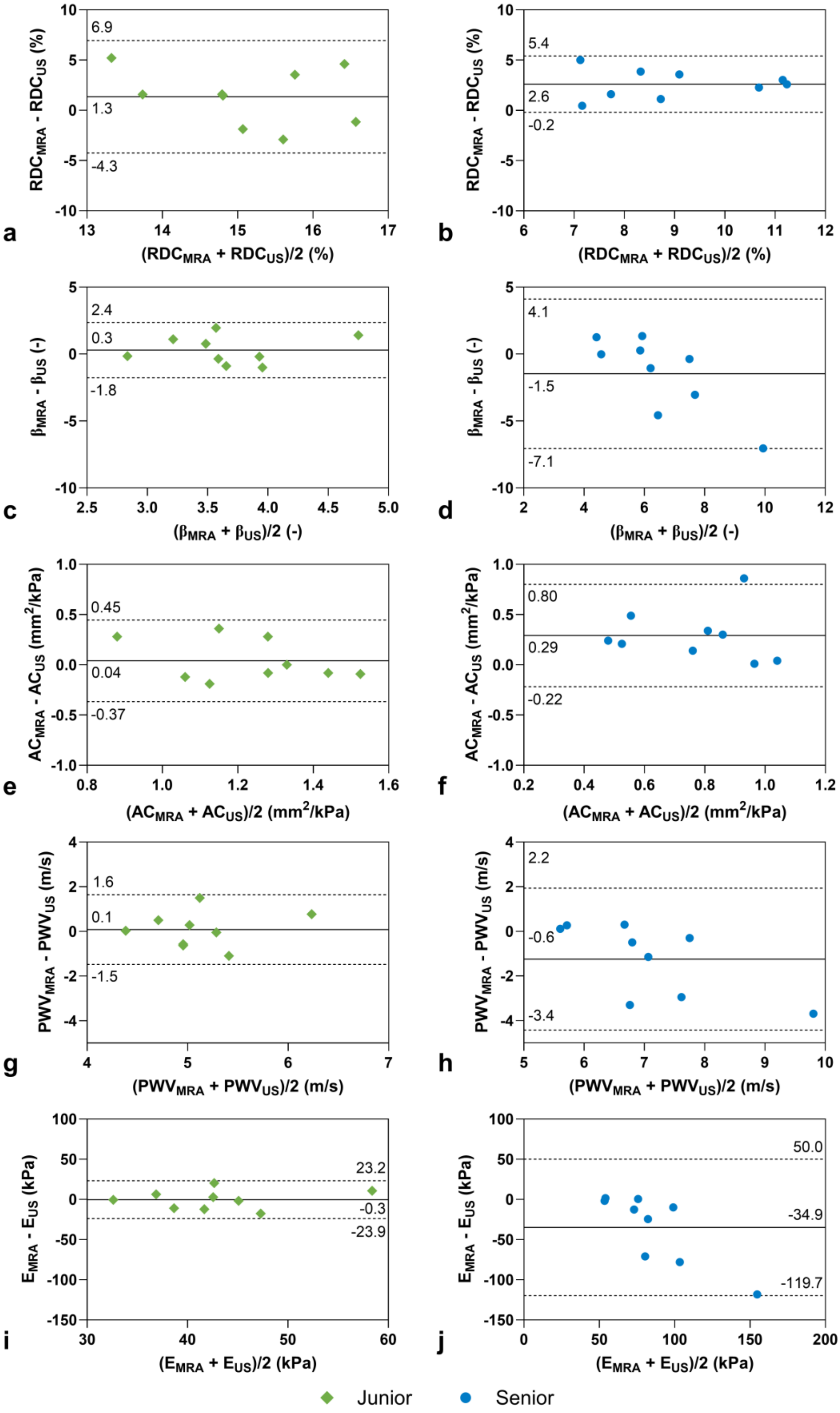

Fig. S4 —Subgroup Bland-Altman plots for the stiffness parameters. In general, narrower confidence intervals and lower biases are observed for the junior cohort (green diamonds).
